# Alcohol use disorder with comorbid anxiety disorder: a case report and focused literature review

**DOI:** 10.1186/s13722-022-00344-z

**Published:** 2022-11-08

**Authors:** Victor Mocanu, Evan Wood

**Affiliations:** 1 British Columbia Centre on Substance Use, 400-1045, V6Z 2A9 Howe St, Vancouver, BC Canada; 2 Department of Medicine, University of British Columbia, 2255 Wesbrook Mall, V6T 2A1 Vancouver, BC Canada

**Keywords:** Alcohol, Anxiety, Comorbidity, Dual diagnosis, Synergy, Treatment recommendations

## Abstract

**Background:**

Alcohol use disorder (AUD) and anxiety disorders (AnxD) are prevalent health concerns in clinical practice which frequently co-occur (AUD-AnxD) and compound one another. Concurrent AUD-AnxD poses a challenge for clinical management as approaches to treatment of one disorder may be ineffective or potentially counterproductive for the other disorder.

**Case Presentation:**

We present the case of a middle-aged man with anxiety disorder, AUD, chronic pain, and gamma-hydroxybutyrate use in context of tapering prescribed benzodiazepines who experienced severe alcohol withdrawal episodes during a complicated course of repeated inpatient withdrawal management. After medical stabilization, the patient found significant improvement in symptoms and no return to alcohol use with a regimen of naltrexone targeting his AUD, gabapentin targeting both his AUD and AnxD, and engagement with integrated psychotherapy, Alcoholics Anonymous, and addictions medicine follow-up.

**Conclusion:**

Proper recognition and interventions for AUD and AnxD, ideally with overlapping efficacy, can benefit individuals with comorbid AUD-AnxD. Gabapentin, tobacco cessation, and integrated psychotherapy have preliminary evidence of synergistic effects in AUD-AnxD. Meta-analysis evidence does not support serotoninergic medications (e.g. selective serotonin reuptake inhibitors) which are commonly prescribed in AnxD and mood disorders as their use has not been associated with improved outcomes for AUD-AnxD. Additionally, several double-blind placebo-controlled randomized trials have suggested that serotonergic medications may worsen alcohol-related outcomes in some individuals with AUD. Areas for future investigation are highlighted.

## Introduction

Alcohol use disorder (AUD) is a prevalent health concern with a recent epidemiologic survey of the United States indicating a lifetime AUD prevalence of 29.1% and previous 12 month prevalence of 13.9% [1]. AUD portends an increased risk for diagnosis of a primary anxiety disorder (AnxD) [1], the definition of which, for the purposes of this case report, aligns with the most recent Canadian clinical practice guidelines for treatment of AnxD to include the DSM-IV umbrella of generalized anxiety disorder (GAD), social anxiety disorder (SAD), panic disorder, specific phobia, obsessive-compulsive disorder, and post-traumatic stress disorder (PTSD) [2]. A quarter or more of individuals with AUD qualify for dual diagnosis with a concurrent primary AnxD in cross-sectional studies [3]. Furthermore, individuals with comorbid AUD-AnxD fare worse in terms of severity, treatment response, and rate of relapse for both conditions [3]. Alcohol intoxication, withdrawal, and biopsychosocial consequences of AUD may all contribute to AnxD symptomatology and vice versa, some individuals with a primary AnxD may use alcohol as self-medication. Alternatively, these conditions may share a common neuropathophysiology influenced by environmental factors at the level of brain structures such as the amygdala and neurotransmitters including gamma aminobutyric acid (GABA), endogenous opioids, dopamine, and serotonin, with implications for clinical management [3].

We present a case report of an individual with comorbid AUD-AnxD and review literature for treatment options to aid clinicians in applying best current evidence for such individuals. Literature review consisted of relevant published studies extracted from author collections and PubMed search with no language or date restrictions for combinations of the following terms: alcohol, alcohol use disorder, anxiety, anxiety disorder, comorbidity, dual diagnosis. In addition, reference lists of selected articles were reviewed for eligible and relevant studies. We find that commonly used treatment options for either AUD or AnxD can have a range of positive and potentially negative impacts for AUD-AnxD which requires accurate understanding of evidence-based care in this area.

## Case Report

A middle-aged man with a longstanding but reportedly well managed history of anxiety disorder dating back to childhood presented for medicalized alcohol withdrawal management services. The individual had a workplace injury approximately 10 years prior that resulted in several spine fractures after which his anxiety deteriorated and he developed AUD. Around this time, an SSRI was trialed for AnxD over several months without benefit and ultimately discontinued. He did not endorse a history for a mood disorder, nor had he received a diagnosis as such. He went on to live with chronic pain and AUD-AnxD for half a decade at which point he had an accidental caustic ingestion resulting in an esophagectomy and jejunostomy tube feeding for one year. His AUD-AnxD subsequently deteriorated further resulting in a prescription of benzodiazepines which were taken for approximately one month until the patient experienced rebound anxiety, worsening of his AUD, and the emergence of gamma-hydroxybutyrate (GHB) use in the context of attempting to discontinue benzodiazepines. The individual sought care through an inpatient withdrawal management program where he had a number of repeated admissions complicated by alcohol withdrawal seizures. He was fortunately stabilized and benzodiazepines were tapered off at which point he was taking no other prescribed medications regularly. Prior to discharge, he was informed of AUD and AnxD treatment options. He was keen to engage with psychosocial supports while starting naltrexone 50 mg once daily and gabapentin 600 mg three times daily.

**Fig. 1:**
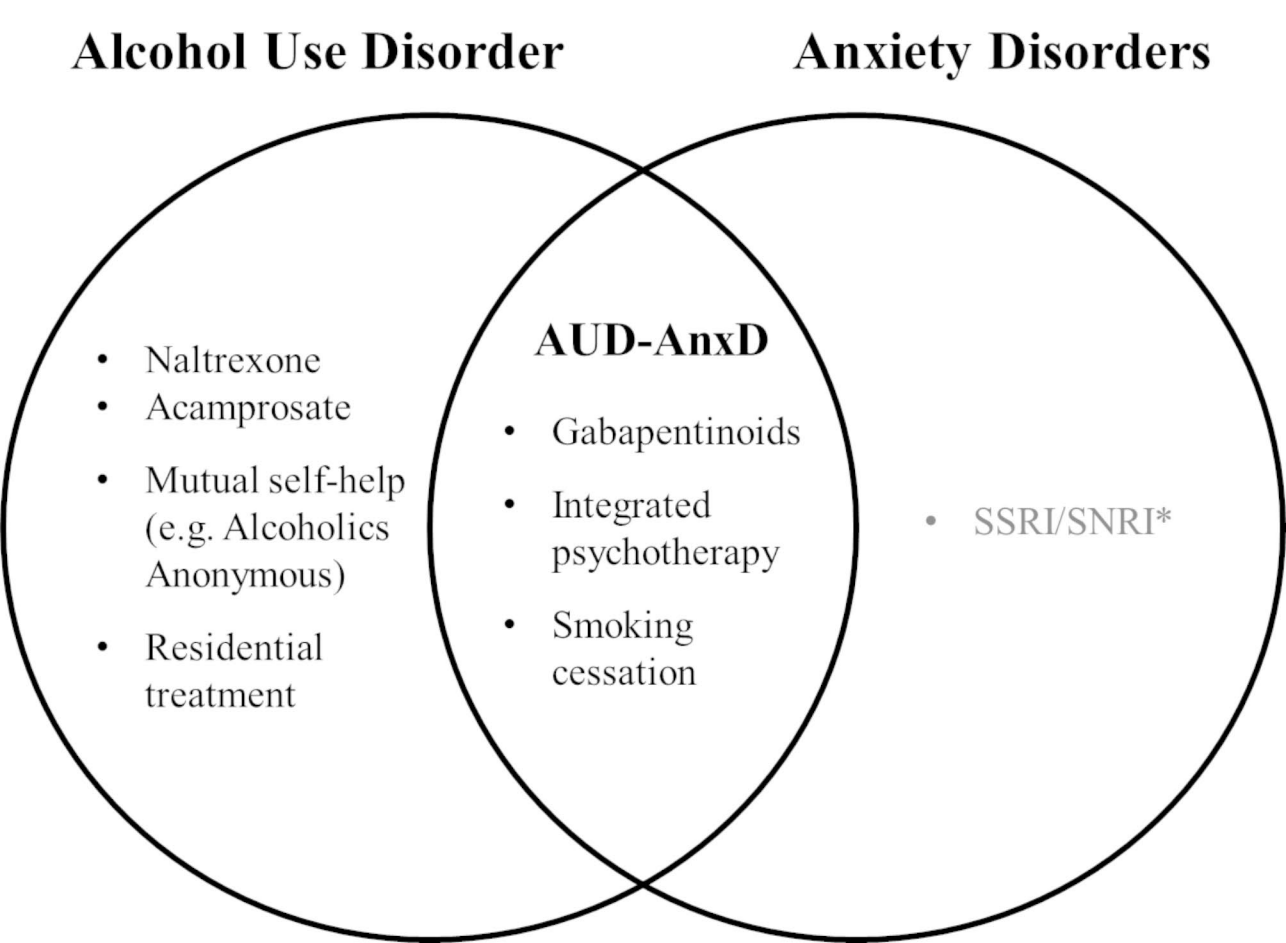
**Venn diagram of current evidence-based interventions reviewed for alcohol use and anxiety disorders** AUD-AnxD indicates comorbid alcohol use disorder and anxiety disorder * Caution to be exercised with serotoninergic agents given evidence for potential worsening of alcohol outcomes in comorbid AUD-AnxD

Eighteen months since discharge, he has found success engaging regularly with substance counselling, Alcoholics Anonymous, and outpatient addictions medicine as well as continuing with naltrexone and gabapentin, the majority of which were intended as synergistic treatments for AUD-AnxD (Fig. 1). He reports good health and no alcohol use, and although experiencing cravings, he describes feeling a shift from the sensation of “premeditated” or inevitable relapse as he had in previous attempts at reducing alcohol use.

### Patient perspective

Hi, I’m here to share with you a little bit of the trial and tribulations endured over the many years of addiction. I was an individual who had a seemingly normal life, but under this was a person with undiagnosed mental problems. I was always a drinker who could take or leave drugs, and kept steady employment and had a very loving partner, it wasn’t until an injury in 2009 when my pace with alcohol quickened and I noticed that my consumption was now an everyday affair. Slowly I developed shakes and my day would start with vomiting until I had alcohol of some variety introduced, this continued on for many years and I was now a product of full-blown alcoholism. There now was no job, no partner and very few things that resembled a person who was still an active part of society. I was in and out of hospitals for experiencing multiple seizures and would go weeks with no food or water and begun isolating. I would become so weak physically that I could not move, and would some days awake in with bruises and cuts from either sizing or pass out, I was never sure which. Then I would begin my process of detox recovery house or treatment only to get home and start the vicious cycle again, some time I could stay sober longer if I was using GHB. Nothing would stop my craving and I no longer felt human. I wanted to die… I am proud to inform you that of Dec 12 2020 I have been living a life of sobriety but continue to struggle with cravings, but I use Alcoholics Anonymous as a support and by surrounding like minded people I find myself sober today.

### Alcohol use disorder and anxiety

AUD and AnxD are common clinical concerns with evidence-based treatment options. Clinicians should recognize that when two conditions such as AUD and AnxD co-occur, clinical management may require nuance and differ from an approach taken in the context of an individual disorder. As we discuss below, research specifically concerning concurrent AUD-AnxD is still rare despite its high prevalence so data must often be extrapolated. The best available evidence indicates that approaches for treating one disorder may be efficacious, ineffective, or in fact counterproductive for comorbid AUD-AnxD.

### AUD treatments

It is critical to recognize and offer proper treatment for AUD even when the primary presenting concern of a client may be an AnxD and vice versa. Whether sequential treatment of AUD and then AnxD, or vice versa, could reduce polypharmacy and iatrogenic harms versus their concurrent treatment remains largely unexplored. Unhealthy alcohol use and AUD are associated with more severe AnxD and conversely abstinence from alcohol is associated with improvement of AnxD [3, 4]. To that effect, longitudinal strategies to address AUD may improve AnxD by extension. As conveyed by the reflections of the individual described in the present report, motivational enhancement, formal psychotherapy, mutual self-help interventions such as Alcoholics Anonymous, and supportive settings such as residential treatment facilities, all highlighting the health benefits of reduction or cessation of alcohol use, can serve an important psychosocial role alongside appropriate evidence-based AUD pharmacotherapy [5]. Nevertheless, the question remains whether certain interventions have additional efficacy in AnxD beyond their efficacy for AUD.

#### Naltrexone and Acamprosate

Naltrexone is a first-line evidence-based pharmacotherapy which functions as a non-selective opioid antagonist, reducing the rewarding effects of alcohol use mediated by endogenous opioids and downstream neurotransmitters. Studies have demonstrated efficacy for reducing binge drinking (number needed to treat [NNT] = 12) as well as relapse to any alcohol use (NNT = 20) [5], which motivated our use of naltrexone in the case strongly featuring AUD. To our knowledge, no clinical trial has yet effectively addressed whether naltrexone treatment for AUD can improve a comorbid AnxD, nor an AnxD disorder in isolation.

Acamprosate, which modulates glutamate and GABA signaling disrupted by chronic alcohol use, is another first-line AUD medication effective in preventing relapse to any alcohol use (NNT = 12) [5]. In terms of comorbid AnxD, some preliminary data suggests benefit of acamprosate for augmentation in various AnxD at doses equivalent or lower than those used in AUD [6]. However, as with naltrexone, there is a paucity of data to confidently gauge the effect of these first-line AUD medications on a comorbid AnxD and further research would be valuable in this area.

#### Gabapentinoids

Gabapentinoids such as gabapentin and pregabalin also modulate glutamate and GABA signaling implicated in AnxD and AUD specifically by antagonism of voltage-gated calcium channels. For AUD, gabapentin can be used in the short-term to treat alcohol withdrawal symptoms and in the longer-term to reduce heavy drinking days although efficacy for other endpoints such as reduction in cravings is debated [7]. Importantly, the data suggest that a key element of clinical efficacy of gabapentin is appropriate patient selection, specifically those with more severe AUD and alcohol withdrawal symptoms. For example, a recent positive randomized clinical trial (RCT) of gabapentin in AUD, which excluded any major psychiatric condition besides PTSD, found that patients with less severe withdrawal tended to fare worse numerically on all indices of alcohol use compared to placebo, although not statistically significant [8]. To date, some literature exists to support using pregabalin during alcohol withdrawal but data for longer-term treatment of AUD is very limited [5]. Interestingly, a RCT comparing pregabalin versus naltrexone in subjects with AUD (approximately 15% with comorbid AnxD in each arm) suggested pregabalin was about as effective as naltrexone in terms of AUD outcomes and more effective in reducing phobic anxiety, a subscale of the particular psychiatric questionnaire used in the study [9]. Further research is needed in this area to better characterize the efficacy of gabapentinoids for treating AnxD specifically in individuals with AUD-AnxD.

The available evidence more strongly supports efficacy of gabapentinoids in treatment of isolated primary AnxD. Gabapentin may be effective for several types of AnxD, [2] although to our knowledge there is no high-quality data yet available to support gabapentin for treatment of GAD which is frequently encountered in clinical practice. On the other hand, pregabalin has extensive data demonstrating efficacy for GAD as well as SAD, so much so that national practice guidelines currently view pregabalin as a first-line agent for these two conditions [2].

In sum, the current evidence indicates gabapentinoids have a role in the treatment of both AUD and AnxD and this class of medications may hold promise for treatment of comorbid AUD-AnxD. This emerging evidence supported the prescription of gabapentin in the above case which we believe ultimately contributed to the substantial benefit the individual found with the regimen described. In the interim while further evidence accumulates, a pertinent consideration in the application of gabapentinoids remains the risk of misuse. Gabapentin misuse occurs in the general population, particularly those with substance use disorders, for a variety of reasons including self-medication (e.g. anxiety, pain, and drug withdrawal symptoms) as well as documented recreational use or self-harm [10]. Even in context of supportive evidence, clinicians should nevertheless be aware of these reports and potential for gabapentinoids to be misused or diverted in treatment of AUD, AnxD, or overlap AUD-AnxD.

#### Other studied AUD medications

Other medications which have received research attention for AUD include baclofen, topiramate, antipsychotics, and benzodiazepines [5]. The evidence base for these agents is even more limited and inconclusive in the realm of AUD and comorbid AUD-AnxD. For example, baclofen is a GABA-B agonist medication with a Cochrane systematic review finding conflicting evidence for AUD, comorbid anxiety, and lack of high-quality evidence for use in alcohol withdrawal [11].

### AnxD pharmacotherapies

#### Psychotropics

Regarding selective serotonin reuptake inhibitors (SSRIs), the prevalent pharmacologic treatment for AnxD [2], a Cochrane systematic review of RCTs for comorbid AUD-AnxD found modest improvements in anxiety measures but unreliable or even unhelpful effects on comorbid AUD [12]. At this juncture, we acknowledge SSRIs are also frequently prescribed according to clinical practice guidelines for treatment of mood disorders that can be challenging to distinguish clinically from AUD and AnxD, which are commonly comorbid [13]. Nevertheless, meta-analyses demonstrate a lack of benefit of SSRIs in the context of AUD with mood disorder with similar concerns of worsening outcomes [14–16]. Given the common co-presentation of anxiety and depression among individuals with AUD, the following discussion instead explores the literature from the perspective of AUD outcomes for individuals with any exposure to SSRI or other serotoninergic medications, such as the individual in our case report, regardless of the primary indication for their use. Notably, comorbid mood and anxiety disorders are frequently reported in the available studies and often served as the primary indication for SSRI use.

We note with interest the underappreciated phenomenon of enhanced serotoninergic neurotransmission exacerbating AUD for certain individuals. For example, a secondary analysis of a RCT studying naltrexone for individuals with AUD and comorbid mood and anxiety disorders found prescriptions for SSRI outside of study protocol during follow-up to be associated with worse drinking outcomes for participants randomized to placebo, an effect which was attenuated if participants received naltrexone [17]. More convincing evidence supporting the potential for harm also exists in several RCTs. In an RCT of the SSRI citalopram for AUD in which mood, AnxD, and both disorders were highly comorbid, participants consumed more alcohol on more days compared to placebo regardless of mood or anxiety measures [16]. Several trials have also evaluated sertraline in AUD and found that younger participants with more severe AUD in particular tend to fare worse when prescribed an SSRI [18], moderated in part by the presence of an allele of the serotonin transporter gene favoring greater serotonergic tone [18]. However, these data are complicated to apply in clinical practice since the predisposing allele may also be found in older participants who happen to develop AUD later in life. Based on allele prevalence in the general US population, Kranzler et al. extrapolated that approximately double the number of individuals with AUD on a population level would be adversely affected by unselected SSRI treatment rather than find benefit [18]. One small trial comparing combinations of venlafaxine and cognitive behavioral therapy (CBT) for AUD found the only effective treatment for reducing heavy drinking days to be CBT alone with placebo medication, suggesting that selective serotonin and norepinephrine reuptake inhibitors such as venlafaxine may likewise be unhelpful for AUD outcomes [19].

Trazodone, another psychotropic medication which modulates serotonin transmission, although used for mood disorders and insomnia rather than AnxD, should be addressed in context of AUD. Trazodone is occasionally prescribed with intent as an antidepressant and sleep aid for individuals with AUD but one RCT found a minimal short-term improvement in sleep quality, while also reporting reduced abstinent days, and increased severity of alcohol consumption following withdrawl of trazodone [20].

In sum, despite the evidence for first-line treatment of isolated AnxD or mood disorders with serotoninergic agents such as SSRIs, clinicians should reconsider and exercise caution in prescribing these medications for patients with comorbid AUD given current observational, RCT, and meta-analysis evidence demonstrating lack of benefit and potential of harm to alcohol use outcomes. In the interim, further investigation is needed to clarify which selected subpopulations in clinical practice would benefit from step-wise approach later involving serotoninergic agents or conversely serotonin antagonists [21]. Careful use of pharmacologic agents targeting AnxD, such as pregabalin or gabapentin, in tandem with other biopsychosocial strategies may have more of an evidence-based role to play since AnxD are frequently comorbid, have been reported to more frequently precede rather than follow AUD [3], and are associated with worsening of AUD [22], suggesting that effective treatment of AnxD may prevent AUD deterioration.

### Tobacco cessation

Although not directly relevant to the individual described in our case report, tobacco use may impact clinical outcomes for individuals with AUD-AnxD. Research indicates that weekly smoking in youth is a risk factor for worsening of AUD [22], and tobacco cessation has been shown to improve anxiety symptoms for both individuals with and without psychiatric disorders [23]. For instance, a recent meta-analysis demonstrated that the nicotinic receptor partial agonist varenicline was safe and conferred a 25% lower risk of anxiety compared to placebo - likely a result of reduction and cessation in tobacco use [24]. However, data specific to AUD-AnxD clinical outcomes after tobacco cessation interventions is still limited and inconclusive to date [25]. In addition to other known broad health benefits such as reduced incidence of cardiovascular disease and cancer, the cessation of tobacco use may also play a synergistic role in treatment of AUD-AnxD.

### Psychotherapies

Formal psychotherapy holds promise for addressing the propagating psychosocial factors of AUD and AnxD alone or in addition to pharmacotherapy. In the realm of AnxD, CBT can be equally or more effective than medication alone [2]. For comorbid AUD-AnxD, motivational interviewing and CBT are effective for both conditions, especially as longer-term modalities with more durable efficacy [26]. Such targeted and integrated psychotherapy might be particularly effective for individuals with AnxD attempting to self-medicate anxiety symptoms by using alcohol.

### Teaching points

AnxD and AUD are prevalent health concerns which frequently co-occur and can compound one another as demonstrated in our report. Their co-occurrence requires a thoughtful approach to treatment given the evidence that treatments of one disorder have been shown to be ineffective or counterproductive for the other disorder (Fig. 1). Although evidence is scarce for synergistic treatments of comorbid AUD-AnxD, the literature supports overlapping efficacy of tobacco cessation, use of gabapentinoids, and integrated psychotherapy, the latter two of which were applied with success for the individual in this case report. While the first-line AUD pharmacotherapies naltrexone and acamprosate do not yet have evidence of concurrent benefit for AnxD, effectively treating AUD may yield downstream benefit for AnxD as suggested by treatment response in this report. Lastly, caution should be exercised with serotoninergic medications such as SSRIs as there appears to be underappreciated evidence suggesting that SSRIs are not of benefit in the context of AUD [12], and may actually worsen alcohol related outcomes for certain individuals [16]. Further investigation is needed to understand synergistic versus step-wise approaches to treatment of comorbid AUD-AnxD and identification of specific population subgroups which may derive benefit with serotoninergic agonists versus antagonists.

## Data Availability

Not applicable to case report.

## Abbreviations

AnxD: anxiety disorder
AUD: alcohol use disorder
AUD-AnxD: comorbid alcohol use disorder and anxiety disorder
CBT: cognitive Behavioral Therapy
GABA: gamma aminobutyric acid
GAD: generalized anxiety disorder
GHB: gamma-hydroxybutyrate
PTSD: post-traumatic stress disorder
RCT: randomized clinical trial
SAD: social anxiety disorder
SSRI: selective serotonin reuptake inhibitors
